# A model for soybean inflorescence architecture based on morphological and gene expression analysis

**DOI:** 10.1007/s00425-025-04864-1

**Published:** 2025-11-13

**Authors:** Francisca Pozo-Muñoz, Ana Berbel, Fanjiang Kong, Francisco Madueño

**Affiliations:** 1 Instituto de Biología Molecular y Celular de Plantas, CSIC-UPV, Campus de Vera, 46022 Valencia, Spain; 2 Guangdong Provincial Key Laboratory of Plant Adaptation and Molecular Design, Innovative Center of Molecular Genetics and Evolution, School of Life Sciences, Guangzhou University, Guangzhou, 510006 China

**Keywords:** Soybean, Inflorescence architecture, Compound inflorescence, Meristem identity genes, *Dt2*, MADS-box genes

## Abstract

**Main conclusion:**

Soybean inflorescence architecture is controversial and regulation of its development unresolved. Our study provides an integral view of its architecture and critical information on the gene network controlling its development.

**Abstract:**

Inflorescence architecture is a highly important trait depending on the arrangement and number of flowers in the inflorescence stem. It strongly contributes to plant morphological diversity, and since it determines the number of flowers and fruits, it has strong potential to influence crop yield. Soybean (*Glycine max*) is a highly relevant grain crop. However, despite many studies involving soybean inflorescence, no clear descriptions of its architecture are available, and the information on this question is controversial. In addition, though a model for the gene network controlling inflorescence meristem identity is established for other legumes, such as pea (*Pisum sativum*) or *Medicago truncatula*, regulation of soybean inflorescence development is not resolved, with the nature of the gene specifying I2 meristem identity not clear yet. Here, we use macroscopic and microscopic observation to analyze soybean inflorescence architecture and RNA in situ hybridization to study the expression of the meristem genes *DT1*, *DT2* and *GmAP1a*, to analyze the control of soybean inflorescence development. Our data demonstrate that, as pea and Medicago, soybean has a compound inflorescence, with flowers formed in secondary inflorescences (I2), and suggest that it is a compound raceme. Our expression study supports that *DT1* and *AP1* specify the identity of I1 and floral meristems, respectively. Importantly, the specific expression of *Dt2* in I2 meristems strongly indicates I2 meristem identity specification by *Dt2* and conservation of the inflorescence gene regulatory network with other legumes. Our study fills an important gap, providing an integral view of soybean inflorescence architecture and novel critical information on the gene network that controls its development.

**Supplementary Information:**

The online version contains supplementary material available at 10.1007/s00425-025-04864-1.

## Introduction

The architecture of the inflorescence, the part of the plant where flowers are formed, is a relevant trait in plants. On the one hand, the great variety of inflorescence architectures very much contributes to the diversity of forms found in angiosperms. In addition, most importantly, inflorescence architecture determines the number of flowers and fruits produced, therefore having a high potential to influence fruit and seed yield.

A main division exists between simple and compound inflorescences. In plants with simple inflorescences, such as Arabidopsis, flowers are directly produced by the shoot apical meristem (SAM) and, therefore, appear at the primary inflorescence (I1) axis. In contrast, in compound inflorescences flowers are formed by secondary or higher order inflorescence meristems, therefore appearing at secondary (I2) or higher order axes, as for instance in pea (Weberling 1989a, 1989b; Supplementary Fig. S1).

**Fig. 1:**
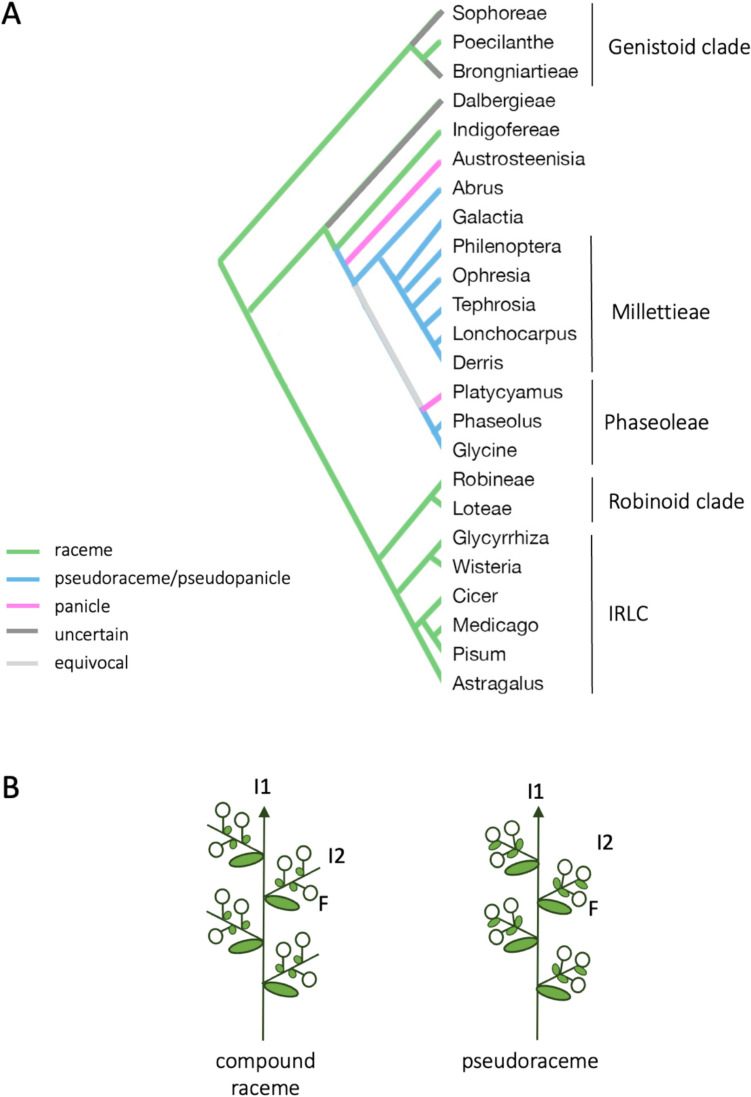
Inflorescence type in different legume clades. **A** Papilionoid legume phylogeny versus inflorescence types (Benlloch et al. 2015; Choi et al. 2022; Hu et al. 2000). **B** Diagrams of compound raceme and pseudoraceme inflorescences. I1, primary inflorescence; I2, secondary inflorescence, F, flower; arrows, meristems; large green ovals, leaves; small green ovals, bracts; closed circles, flowers

The basic inflorescence type in legumes is the compound, a.k.a. double, raceme (Fig. 1; Endress 2010; Weberling 1989b). In this type of compound inflorescence, lateral inflorescences are racemes, where internodes are developed, the flowers have pedicels and usually open in the order in which they are produced (Endress 2010; Weberling 1989a, 1989b). Nevertheless, other types of compound inflorescences are also found in legumes. Thus, while in species from Robineae, Loteae and the IRLC clades, which include pea (*Pisum sativum*) and *Medicago truncatula*, the most frequent inflorescence type is the compound raceme (Fig. 1A; Supplementary Fig. S1; Benlloch et al. 2015; Hu et al. 2000), Phaseoleae species, which include common bean (*Phaseolus vulgaris*) and soybean (*Glycine max*), have been described as mostly having pseudoracemes (Fig. 1A; Hu et al. 2000). In contrast to the lateral inflorescences of compound racemes, where flowers are solitary at each node and commonly arranged helically (Fig. 1B; Tucker 1987), in lateral inflorescences of pseudoracemes, the flowers appear with different patterns from that one, frequently in triads, and some of the flowers emerge from the same node (Fig. 1B; Tucker 1987).

Soybean is a very important grain crop, being the source for about one quarter of plant proteins for human and animal consumption and the supply for more than half of the food oil of the world (Graham and Vance 2003). In addition, soybean has become an excellent model for legume molecular genetics. Genetic transformation works well in soybean (Lu et al. 2017) and this, together with a high-quality reference genome (Schmutz et al. 2010), currently allows efficient functional genomic approaches, including CRISPR and ChIPseq (Bu et al. 2021; Lin et al. 2022). This is leading to many studies in different fields, such as the regulation of flowering time  and plant and inflorescence architecture, all of which are critical for the improvement of yield (Wang et al. 2024; Zhao et al. 2024).

However, despite being such an important species, not a proper description of soybean inflorescence development is available. There are some studies on the topic in the literature, but they are usually partial and there are significant discrepancies between them. In some cases, soybean has been represented as having a simple inflorescence, with flowers directly appearing in the leaf axils of the primary inflorescence stem (Liu et al. 2016; Zhang et al. 2019). More frequently, soybean inflorescence is described as a compound one; but, while in some cases its inflorescence is presented as a compound raceme (Li et al. 2024; Zhong and Kong 2022), in others it has been described as a pseudoraceme (Hu et al. 2000). To clarify these discrepancies, we think that a systematic description of soybean inflorescence architecture would be useful.

The architecture of each inflorescence depends on the network of regulatory genes that control the identity and development of the meristems in the inflorescence apex. The basic gene network for simple inflorescences like Arabidopsis is well established. *TERMINAL FLOWER 1* (*TFL1*), coding a regulatory protein from the phosphatidylethanolamine-binding protein family, maintains the identity of the primary Arabidopsis inflorescence (I1) meristem. *TFL1* is expressed in the I1 meristem, preventing the expression of floral genes in this domain. This precludes the conversion of the I1 meristem into a floral meristem and allows its indeterminate growth (Benlloch et al. 2007; Bradley et al. 1997). Conversely, floral identity genes, such as *APETALA1* (*AP1*) and *LEAFY* (LFY), coding transcription factors, are expressed in the primordia at the flanks of the I1 meristem, conferring them floral meristem identity. Their expression there also prevents the expression of *TFL1* in these meristems and their conversion into inflorescence-like structures (Benlloch et al. 2007; Mandel et al. 1992; Weigel et al. 1992).

The gene network controlling the specification of meristem identity in the compound inflorescence of papilionid legumes is also quite well established for pea and *Medicago truncatula*, and shares elements with that of Arabidopsis. Thus, the identity of the I1 meristem is maintained by *TFL1*-like genes, *DETERMINATE* (*DET*)/*PsTFL1a* in pea and *MtTFL1* in Medicago (Cheng et al. 2018; Foucher et al. 2003). As in Arabidopsis *TFL1*, both genes are expressed in the I1 meristem and their mutation leads to plants where the I1 becomes determinate, forming a terminal inflorescence (Berbel et al. 2012; Cheng et al. 2018). Also as in Arabidopsis, floral meristem identity is specified by *LFY* homologues and by the *AP1* homologues *PROLIFERATING INFLORESCENCE MERISTEM* (*PIM*)/*PsAP1* in pea and *MtPIM/MtAP1* in Medicago (Benlloch et al. 2006; Berbel et al. 2001; Cheng et al. 2018; Hofer et al. 1997; Taylor et al. 2002). As in Arabidopsis *LFY* and *AP1*, those legume genes are expressed in floral meristems and their mutations lead to plants where flowers exhibit inflorescence-like traits (Cheng et al. 2018; Hofer et al. 1997; Taylor et al. 2002). Finally, the identity of I2 meristems is specified by genes which encode MADS-BOX transcription factors, *VEGETATIVE1* (*VEG1*)/*PsFULc* in pea and *MtFULc* in Medicago, with similarity to Arabidopsis *FRUITFUL* (*FUL*) and *AGL79*, belonging to the EuFULII clade of the AP1/FUL subfamily (Berbel et al. 2012; Cheng et al. 2018). *VEG1/PsFULc* and *MtFULc* are expressed in the I2 meristems of the inflorescence and are required for these meristems to develop into I2. In *psfulc* and *mtfulc* mutants, I2 meristems are transformed into I1 meristems and stay vegetative, never forming flowers or floral organs (Berbel et al. 2012; Cheng et al. 2018). Similar to Arabidopsis, the expression domains of the *TFL1*, *FULc* and *AP1* genes in the pea and Medicago inflorescence apices are maintained through a network of mutual transcriptional repression (Berbel et al. 2001; Benlloch et al. 2015; Cheng et al. 2018).

In soybean, the role in inflorescence development of a *TFL1*-like gene, *Dt1*, and of *AP1*-like genes, *GmAP1s*, is well established and is similar to those of their pea and Medicago homologs. *Dt1* is essential for the indeterminate growth of the I1 meristem, and most likely for its identity (Liu et al. 2010; Tian et al. 2010; Yue et al. 2021), and *GmAP1* genes regulate processes such as flower development and flowering and likely also floral meristem identity (Chen et al. 2020; Yue et al. 2021). Nevertheless, the nature of the gene specifying I2 meristem identity is not clear yet.

The soybean *Dt2* gene has high similarity with the pea *VEG1/PsFULc* and the Medicago *MtFULc* genes, and therefore is a good candidate for the soybean I2 meristem identity gene. However, though *Dt2* has been much studied, and it is known to control inflorescence semi-determinacy, flowering and branching (Liang et al. 2022; Ping et al. 2014), so far it has not been connected with the development of I2 shoots. Further studies are required to assess a possible role of *Dt2* in soybean I2 meristem identity.

In this study, we present a detailed morphological description of the soybean inflorescence that shows that it is a compound inflorescence, where flowers are produced in axillary I2s, with an architecture compatible with that of a compound raceme. We also analyze the expression of soybean homologs of the genes that are essential for the identity of the meristems in the inflorescence in other legumes. Our results suggest that *Dt2* is involved in the specification of the I2 meristem and that the genetic network controlling soybean inflorescence architecture is conserved with that of pea and Medicago.

## Results

### Description of the development of the soybean plant

To present the plant architectural context in which soybean inflorescence is formed, we first studied the development of indeterminate soybean plants (genotype Williams 82), from germination to formation of the inflorescence, flowers and pods, under our growing conditions (long days, 16-h light/8-h darkness).

**Fig. 2:**
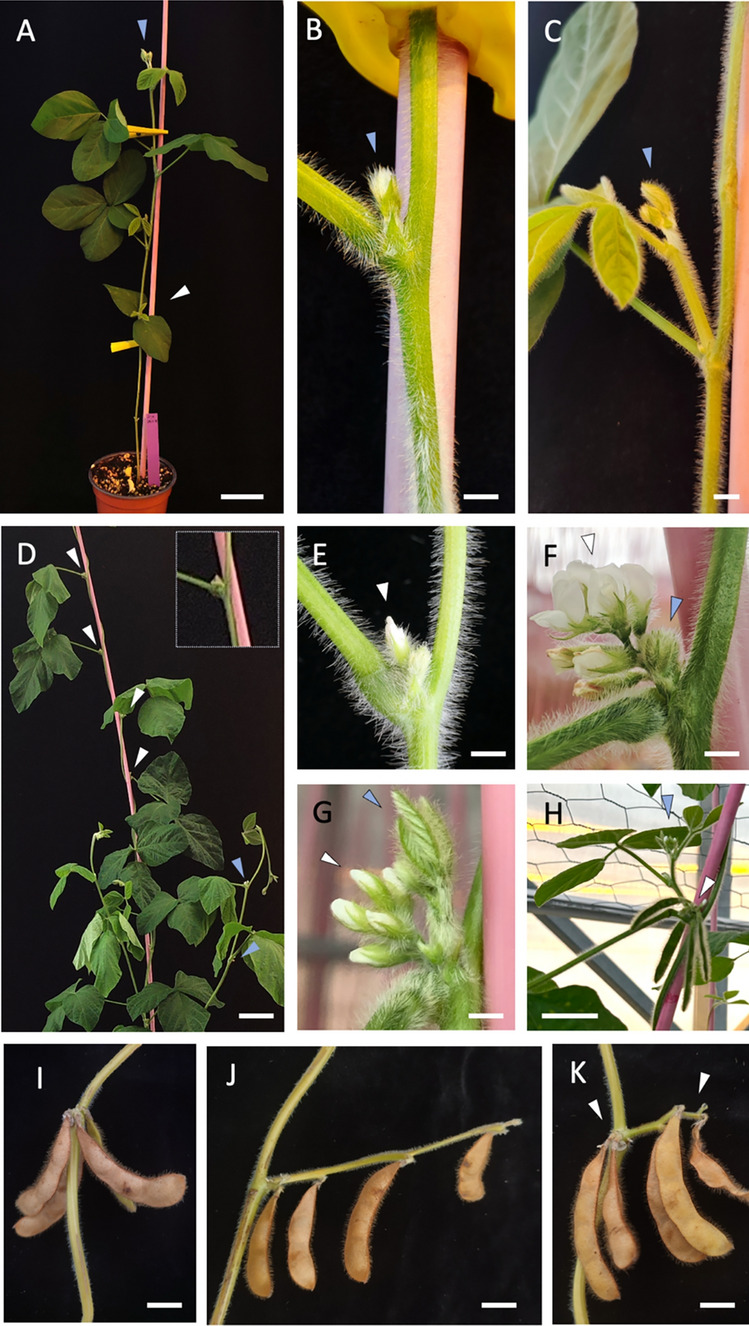
Development of the soybean plant and inflorescence. **A** Plant at the vegetative phase, with the basal pair of opposite unifoliate leaves, marked with a white arrowhead, and four expanded trifoliate leaves. Blue arrowhead marks the shoot apex. **B** Vegetative bud, marked with a blue arrowhead, in the axil of a leaf. **C** Young axillary branch. The branch shoot apex is marked with a blue arrowhead. **D** View of a plant at the inflorescence phase, after floral transition. White arrowheads mark axillary secondary inflorescence (I2) buds in the primary inflorescence (I1) stem. Blue arrowheads mark inflorescence buds formed in a branch. **E**–**G** Axillary I2s at different developmental stages. Blue arrowheads mark accessory vegetative buds. **H** White arrowhead marks an axillary I2 whose flowers have already formed pods. The blue arrowhead marks the apex of an accessory branch associated with that I2. **I**, **J** Soybean pods showing different types of arrangements. White arrowheads in K mark two groups of pods that might derive from two independent I2s in the same node. Scale bars: **A**, **D**, **H**, 5 cm; **B**, **C**, **E**, **F**, **G**, 2 mm; **I**, **J**, **K**, 2 cm

During the vegetative phase, soybean plants consist of an elongated stem bearing leaves (Fig. 2A) whose axils contain vegetative buds that are initially dormant (Fig. 2B). At a certain point, these buds begin to grow out, giving rise to branches (Fig. 2C).

The leaf axils of the nodes produced in the reproductive phase, after floral transition, contain inflorescence buds (Fig. 2D–G). These axillary buds produce short inflorescences with a few flowers, which appear to be formed sequentially (Fig. 2F, G; Supplementary Fig. S2A). Frequently, in the same leaf axil, a supernumerary vegetative bud is formed (termed accessory bud; Grbić and Bleecker 2000), appearing adjacent to the axillary inflorescence (Fig. 2F), such that accessory branches develop from the same leaves that subtend the inflorescences (Fig. 2G, H).

Upon formation of the pods, little elongation of the internodes between them is generally observed (Fig. 2I, H, K). However, in some cases, internodes elongate leading to structures that may resemble racemes (Fig. 2J). Occasionally, pods appear distributed into two separate clusters, the distal one perhaps derived from an accessory branch (Fig. 2K).

This macroscopic analysis clearly shows that soybean has a compound inflorescence with secondary inflorescences (I2) appearing at the axils of the leaves of the primary inflorescence (I1). Furthermore, our results suggest that in these axillary inflorescences, the flowers appear sequentially (Supplementary Fig. S2).

### Analysis by SEM of soybean inflorescence development

To examine in detail the development of the meristems at the inflorescence apex and to assess the pattern of floral emergence in axillary inflorescences, we analyzed developing shoot apices using scanning electron microscopy (SEM).

**Fig. 3:**
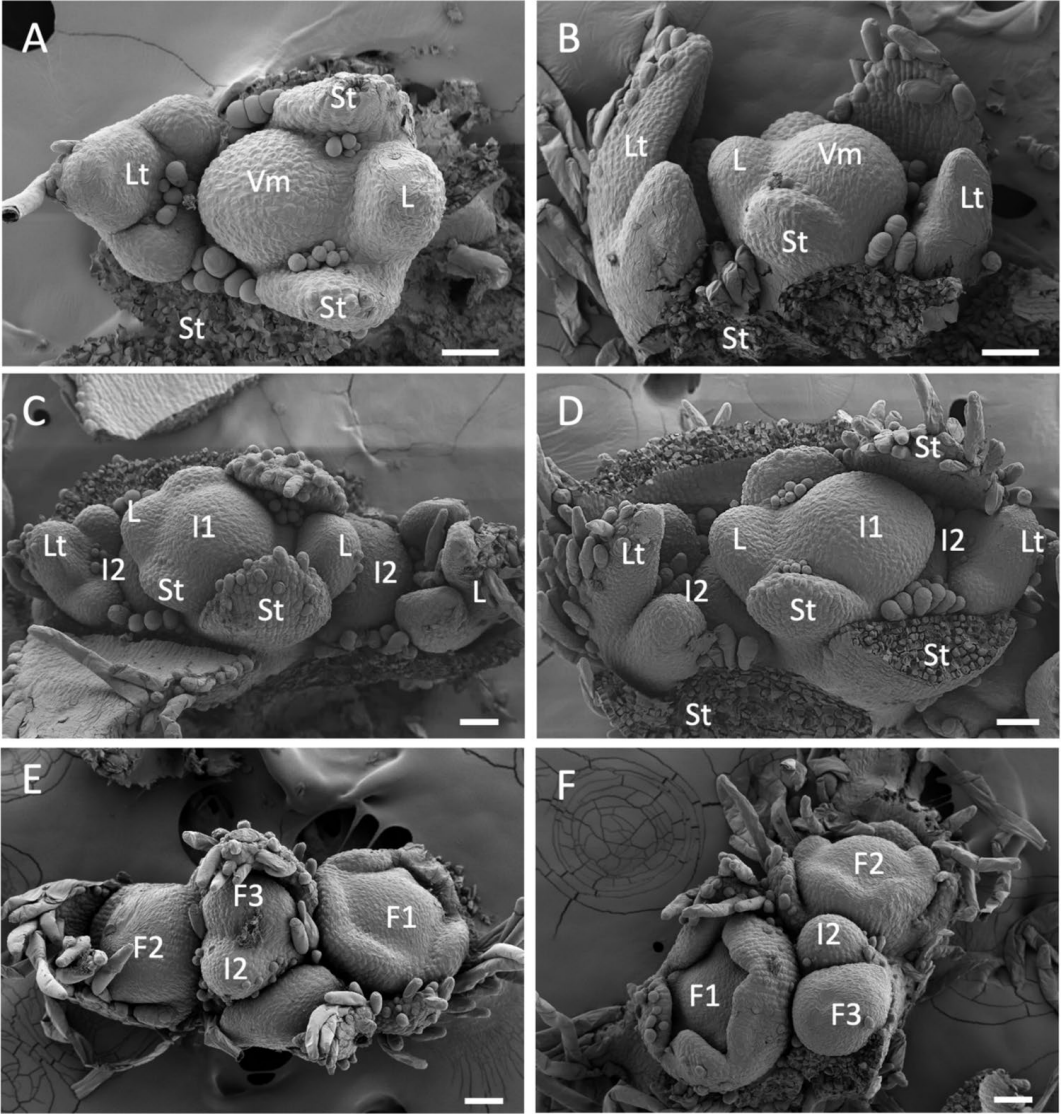
SEM analysis of the development of the soybean vegetative and inflorescence apices. **A**, **B** Vegetative shoot apices from plants with 1 (**A**) or 5 (**B**) expanded trifoliate leaves. **C**, **D** Primary inflorescence apices from plants with 15 expanded trifoliate leaves. **E**, **F** Secondary inflorescences from flowering plants, each one with three floral primordia of different ages. Vm, vegetative meristem; I1, primary inflorescence meristem; I2, secondary inflorescence meristem; St, stipule; L, leaf primordia; Lt, trifoliate leaf primordia; F1 *–*  F3 floral primordia. Scale bars: 50 μm

The shoot apices of vegetative plants consist of a vegetative shoot apical meristem (SAM) flanked by leaf primordia, each of these with two stipule primordia (Fig. 3A, B). The youngest leaf primordia are very small, comparable in size to their stipule primordia. Only in more developed leaf primordia it can be observed that they will form a trifoliate leaf. At this stage, the axils of the leaf primordia appear empty, suggesting that vegetative axillary meristems are not immediately produced after leaf primordia formation (Fig. 3A, B).

Following floral transition, the vegetative shoot apex becomes the apex of the primary inflorescence (I1). SEM images of these apices show that, similar to vegetative apices, I1 apices consist of a central inflorescence SAM flanked by newly initiated single leaf primordia and stipules, as well as by older leaf primordia with trifoliate morphology (Fig. 3C, D). Unlike vegetative apices, meristems—presumably secondary inflorescence (I2) meristems—are observed in the axils of the trifoliate leaf primordia in these I1 apices (Fig. 3C, D). This indicates that in the I1 inflorescence apex, axillary I2 meristems are produced shortly after leaf primordia initiation, in contrast to the delayed appearance in vegetative apices.

SEM analysis of lateral secondary inflorescences showed that the I2 meristems give rise to axillary inflorescences. In each of the developing I2s shown in Fig. 3E, F, three floral buds at different developmental stages (from emerging floral meristems to young floral buds with visible sepal primordia) are observed around the growing I2 meristem (Fig. 3E, F). These data show that the floral buds, apparently arranged helically, are produced sequentially by the I2 meristem.

Taken together, these observations show that soybean flowers are produced by axillary I2 meristems, confirming that soybean has a compound inflorescence (Supplementary Fig. S2). In addition, the data confirm that flowers are formed sequentially by the I2 meristems, following a helical arrangement (Fig. 3E, F), resembling the ontogeny of a raceme (Tucker 1987; Endress 2010), particularly in legume compound racemes.

### Expression of meristem identity genes during development of soybean inflorescence

Our morphological analysis suggests that soybean inflorescence is a compound raceme, similar to those of pea and Medicago. Since the architecture of the inflorescences depends on the network of genes that control the identity and development of their meristems, we used in situ hybridization to analyze the expression of soybean homologs of genes responsible for meristem identity in the inflorescence apex of the pea and Medicago compound racemes (Benlloch et al. 2015; Cheng et al. 2018). On the one hand, we did that because this type of gene are good expression markers for the meristems in the inflorescence apex, I1, I2 and floral meristems, and their expression pattern can help interpret the morphological descriptions. On the other hand, we did that also because comparing the expression patterns of these genes in soybean with those of their homologs in other species can help assess whether the gene network controlling soybean inflorescence development is similar or not to that of other legumes.

We focused on the expression analysis of the *Dt1*, *Dt2* and *GmAP1* soybean inflorescence-related genes (Hou et al. 2024).

*Dt1* is a *TFL1*-like gene in soybean, homolog to pea *DET/PsTFL1a* and to Medicago *MtTFL1*, which specify the identity of the I1 meristem and prevent its determination (Cheng et al. 2018; Foucher et al. 2003). This function seems conserved in *Dt1*, as the soybean *dt1* mutant exhibits a determinate inflorescence phenotype very similar to that of the pea and Medicago *det/pstfl1a* and *mttfl1* mutants (Liu et al. 2010; Tian et al. 2010). *Dt1* expression, as detected by qRT-PCR, is observed in main shoot apices early in development, with floral induction, with its level gradually decreasing with development (Ping et al. 2014). Among the four *TFL1*-like genes present in the soybean genome (Wang et al. 2023), *Dt1/GmTFL1b* has been the most extensively studied. We selected this gene for expression analysis due to its apparent functional conservation with *DET/PsTFL1a* and *MtTFL1*.

**Fig. 4:**
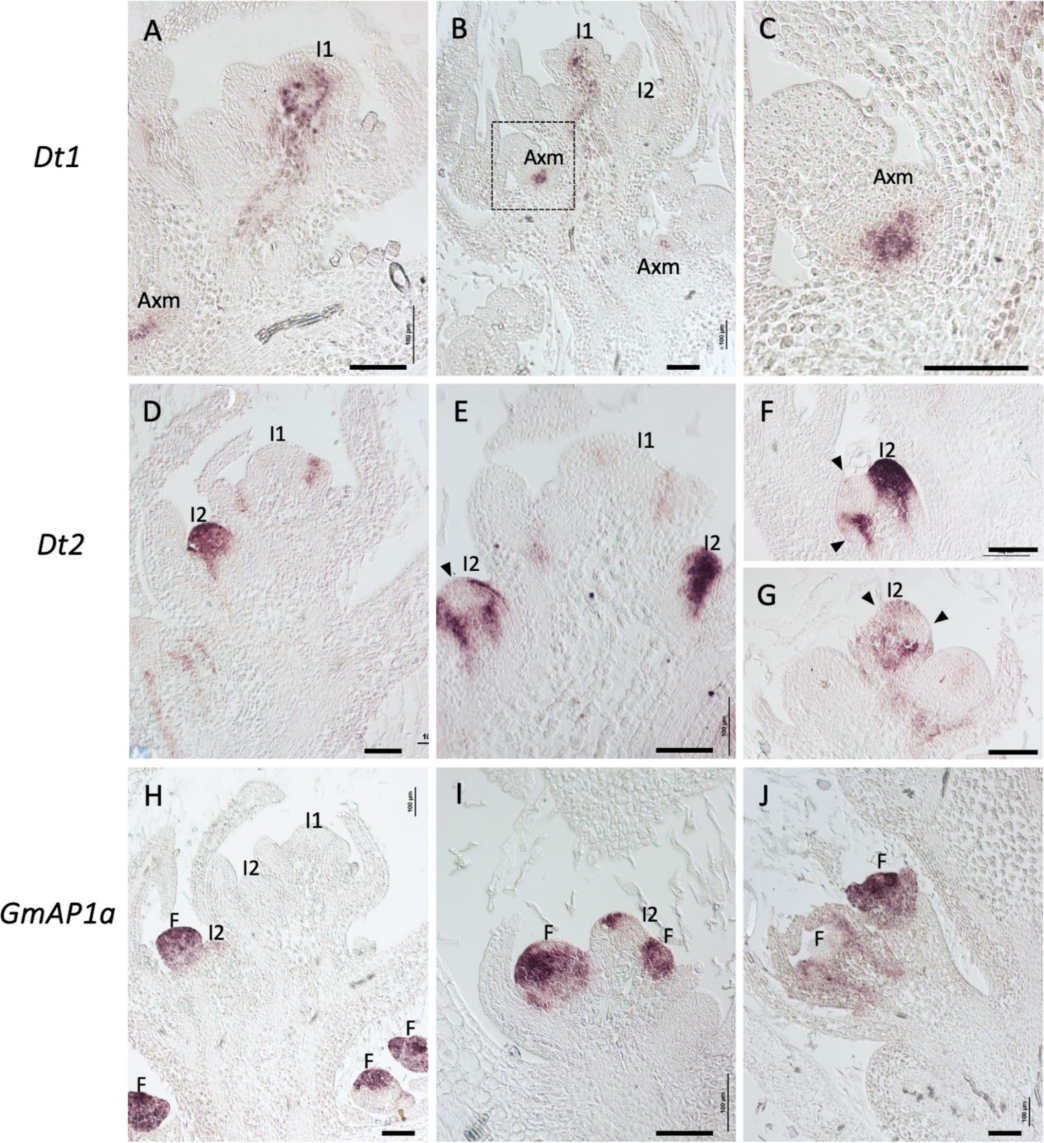
Expression analysis of *Dt1*, *Dt2* and *GmAP1a* genes in soybean inflorescence apices by in situ hybridization. In situ hybridization of inflorescence shoot apices sections from plants with 9 expanded trifoliate leaves. **A**, **B** Inflorescence apex sections hybridized with a *Dt1* probe. **C** Close-up of the image framed in B. **D**–**G** Sections hybridized with a *Dt2* probe. Arrowheads point to areas lacking *Dt2* expression. **H**–**J** Sections hybridized with a *GmAP1a* probe. Axm, vegetative axillary meristem; I1, primary inflorescence meristem; I2, secondary inflorescence meristem; F, floral meristem or floral primordia. Scale bars: 100 μm. Negative controls hybridized with the corresponding sense probes are shown in Supplementary Fig. S4

In our analysis of soybean inflorescence apices, the most conspicuous expression of *Dt1* was observed in the shoot apical I1 meristem (SAM), restricted to the central region of the I1 meristem, and its expression was excluded from the outer L1 and L2 cell layers (Fig. 4A, B). Additional expression was also detected in the stem vasculature beneath the SAM (Fig. 4A, B). In addition, *Dt1* expression was detected in lower axillary positions, likely representing I1 meristems of accessory branches (Fig. 4A–C). The *Dt1* expression pattern in these axillary structures, in the center of the I1 axillary meristem, resembles its expression in the I1 SAM (Fig. 4A–C). The *Dt1* expression pattern is therefore similar to that of *DET/PsTFL1a* and *MtTFL1* and, together with its consistent central expression in I1 and axillary meristems, this further supports the role of *Dt1* in specifying I1 identity.

The soybean *Dt2* gene is a homolog of pea *VEG1/PsFULc* and of Medicago *MtFULc* genes (Supplementary Fig. S3), which in these species specify the identity of the I2 meristems that give rise to the secondary inflorescences (Berbel et al. 2012; Cheng et al. 2018; Ping et al. 2014). *Dt2* expression is also detected by qRT-PCR in shoot apices with floral induction, its level increasing along the flowering process (Ping et al. 2014). In addition to *Dt2* (A.K.A. *GmFUL3b*), there is a second homolog of *VEG1/PsFULc* and *MtFULc* genes, *GmFUL3a* (Cao et al. 2015). However, *Dt2* has been the most characterized and only for *Dt2*, an inflorescence phenotype has been described, as semi-determinate (Ping et al. 2014). Therefore, we selected *Dt2* for expression analysis.

To simplify the nomenclature of legume *FUL* genes, we suggest modifying the naming of the *FUL* soybean genes (Cao et al. 2015), following the naming previously established for the *FUL* genes of other legumes, pea and Medicago. We propose that the names of the *FULc* homologs *GmFUL3a* and *Dt2/GmFUL3b* be changed to *GmFULc1* and *Dt2/GmFULc2*, respectively, and propose renaming, in a similar way, the other four soybean *FUL* genes (Supplementary Fig. S3).

In the inflorescence apex,  in situ  hybridization revealed *Dt2* expression restricted to leaf axils, where I2 meristems form (Fig. 4D, E), not being observed in the I1 meristem. Expression seemed stronger in basal positions, coinciding with I2s in a more advanced developmental stage (Fig. 4D, E). In lower axillary positions, where I2 meristems were developing into axillary inflorescences, areas lacking *Dt2* expression were observed, likely corresponding to regions where floral meristems had initiated (Fig. 4E, F, G). In summary, our results show that *Dt2* is expressed in axillary structures of the inflorescence apex, most likely corresponding to I2 meristems, which suggests a role of *Dt2* in I2 meristem identity specification.

*GmAP1a* gene is a soybean homolog of the *AP1*-like genes *PIM/PsAP1* from pea, and *MtPIM*/*MtAP1* from Medicago, which specify the identity of the floral meristems and organs (Benlloch et al. 2006; Berbel et al. 2012; Taylor et al. 2002). The soybean genome contains four *AP1* genes, likely with partly redundant functions (Chen et al. 2020). We selected *GmAP1a* for our study because it has been shown by in situ hybridization analysis to have a typical *AP1* expression pattern, and its over-expression has been shown to control soybean inflorescence architecture (Chi et al. 2011; Chen et al. 2020).

In soybean inflorescence apices, the expression of *GmAP1a* first appeared at basal axillary positions, associated with emerging I2 meristems (Fig. 4H). In some axillary structures, possibly developing I2 inflorescences, *GmAP1a* expression marked several domes, presumably corresponding to floral meristems (Fig. 4I). In more advanced structures, the expression of *GmAP1a* was evident in floral primordia, particularly in the first and second floral organ whorls (Fig. 4J). These data indicate that the expression of *GmAP1a* in the inflorescence apex marks floral meristem initiation and the formation of floral primordia.

**Fig. 5:**
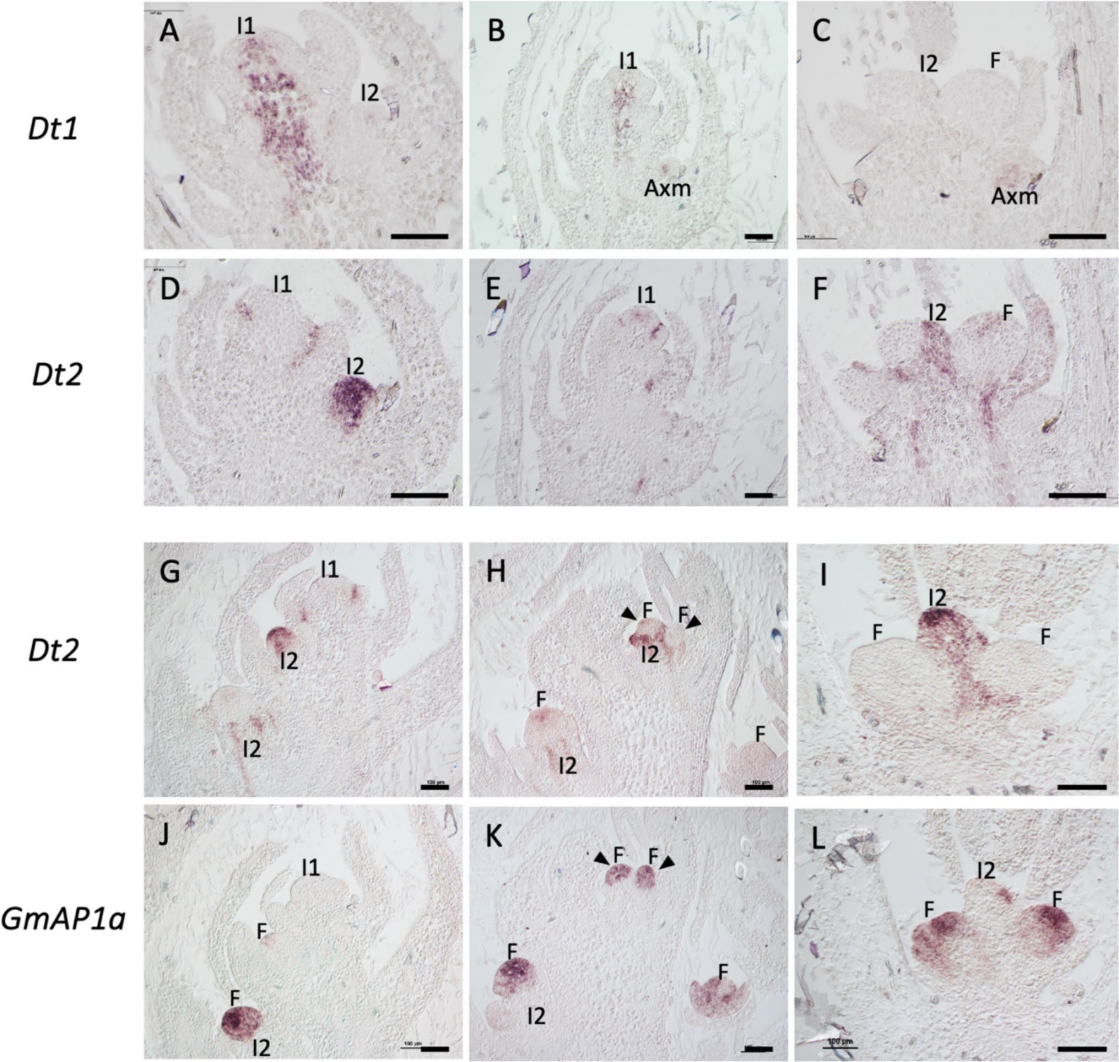
Expression analysis of *Dt1*, *Dt2* and *GmAP1a* genes by in situ hybridization on serial consecutive sections of soybean inflorescence apices. In situ hybridization of inflorescence shoot apices sections from plants with 9 expanded trifoliate leaves. **A**, **B** Sections of two different apices hybridized with *Dt1*. **C** Section of an I2 axillary bud hybridized with *Dt1*. **D**, **E** Sections consecutive to those of the apices in A and B hybridized with *Dt2*. **F** Section consecutive to that in C hybridized with *Dt2*. **G**, **H** Sections of two different apices hybridized with *Dt2.* **I** Section of an I2 axillary bud hybridized with *Dt2*. **J**, **K** Sections consecutive to those of the apices in G and H hybridized with *GmAP1a*. **L** Section consecutive to that in I hybridized with *GmAP1a*. Axm, vegetative axillary meristem; I1, primary inflorescence meristem; I2, secondary inflorescence meristem; F, floral meristem or floral primordia. Scale bars: 100 μm. Negative controls hybridized with the corresponding sense probes are shown in Supplementary Fig. S4

To better compare the expression domains, we performed in situ hybridization on serial consecutive sections of inflorescence apices. In situ hybridization of *Dt1* and *Dt2* showed that they exhibit a clear complementary expression pattern, with *Dt1* being expressed in the I1 meristem and *Dt2* in axillary I2 meristems (Fig. 5A, B, D, E). In more advanced axillary structures, they also showed complementarity; *Dt2* occupied a broader domain, likely representing the I2 meristem, while *Dt1* was confined to a lateral axillary meristem, possibly the bud of an accessory branch (Fig. 5C, F).

Serial sections also revealed a clear complementarity between *Dt2* and *GmAP1a* expression domains. Thus, *GmAP1a* expression was observed in meristems emerging from developing I2s, in domains where *Dt2* expression had disappeared (Fig. 5G, H, J, K). A closer view of a node with a developing axillary structure also showed clear complementarity, with *Dt2* expressed in a central domain, presumably the developing I2, and *GmAP1a* in lateral meristems formed in that I2, presumably floral meristems (Fig. 4I, L).

**Fig. 6:**
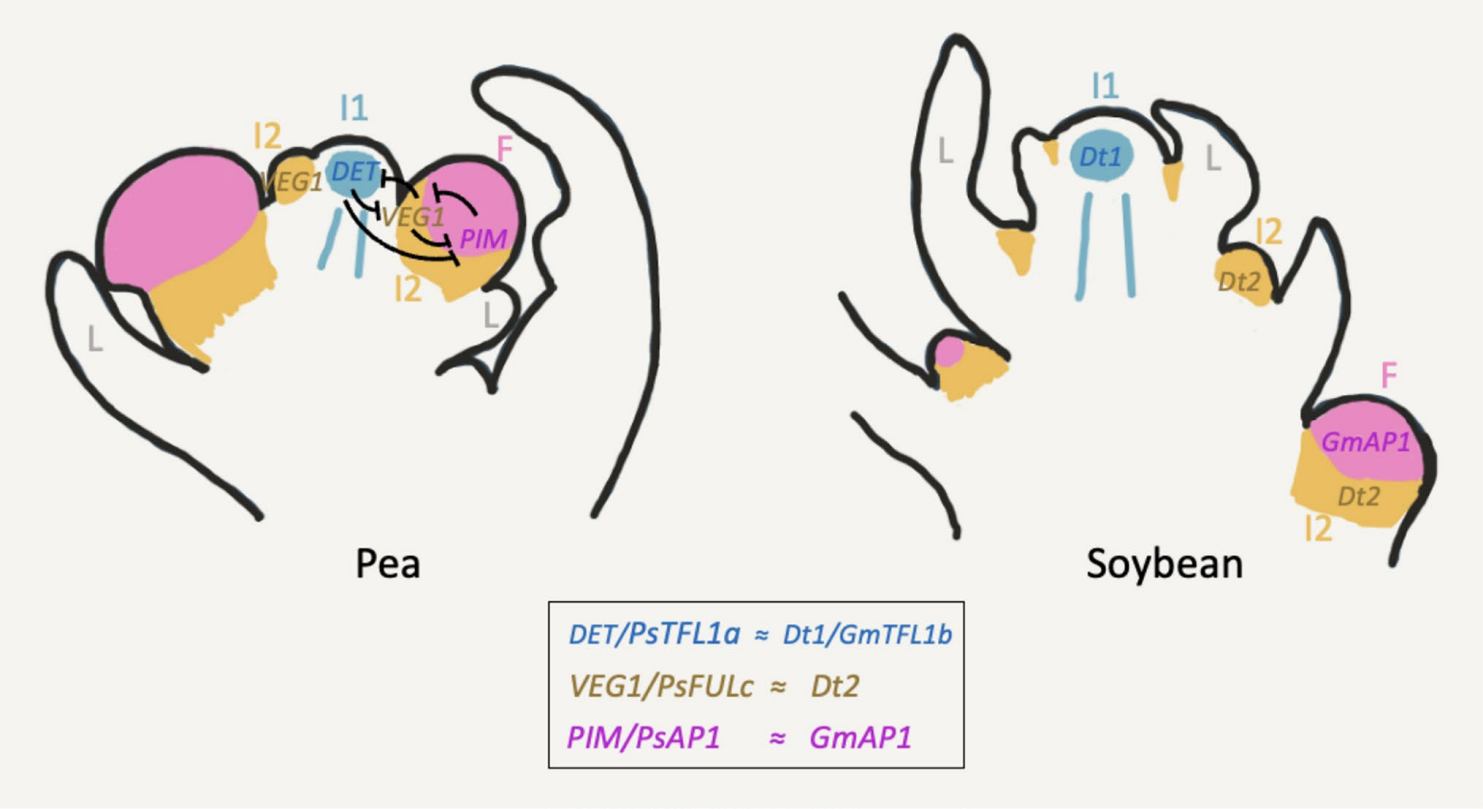
Diagrams of young inflorescence apices of pea and soybean representing the expression of meristem identity genes controlling the identity of the meristems in the inflorescence and genetic interaction of meristem genes in the pea inflorescence. Light blue represents the expression of *DET/PsTFL1a* in pea and *Dt1/GmTFL1b* in soybean; light brown represents the expression of *VEG1/PsFULc* in pea and *Dt2/GmFULc2* in soybean; pink represents the expression of *PIM/PsAP1* in pea and *GmAP1* in soybean. Blunt arrow in the pea apex indicates repression. I1, primary inflorescence meristem; *I2,* secondary inflorescence meristem;  F, floral meristem; L, leaf

In summary, our results show that *Dt1*, *Dt2* and *GmAP1a* define distinct and complementary meristematic domains in the soybean inflorescence apex: *Dt1* in the I1 meristem, *Dt2* in the I2 meristems and *GmAP1a* in floral meristems (Fig. 6).

## Discussion

In this work, we analyzed the architecture of the soybean inflorescence and studied in detail the expression of the *Dt1*, *Dt2* and *GmAP1a* genes, whose homologs have been shown to play a key role in the regulation of inflorescence development in other legumes, such as pea and Medicago. Our observations demonstrate that soybean has a compound inflorescence, possibly a compound raceme, and, importantly, support that these three genes are central components of the regulatory network controlling the specification of the identity of the meristems in the soybean inflorescence apex.

### Clarifying soybean inflorescence architecture

There is some discrepancy regarding the architecture of the soybean inflorescence, partly because rather limited clear illustrations of the soybean inflorescence are available. Some studies apparently present the soybean inflorescence as a simple inflorescence, with individual flowers being formed in the leaf axils of reproductive nodes (Liu et al. 2016; Zhang et al. 2019). Nevertheless, most reports describe the soybean inflorescence as a compound inflorescence, with secondary inflorescences (I2) in the leaf axils of the primary inflorescence (I1) stem, which produce the flowers (Li et al. 2024; Zhong and Kong 2022).

Our observations, both at the macroscopic level and through SEM, and tissue sections in the in situ hybridizations, clearly show that soybean has a compound inflorescence, with floral meristems formed at axillary I2 meristems.

Previous studies have proposed the pseudoraceme as the inflorescence type in soybean (Hu et al. 2000) though other reports suggest that soybean inflorescence is a compound raceme (Li et al. 2024; Zhong and Kong 2022). Our observations suggest that soybean inflorescence might be a compound raceme, as indicated by the sequential formation of flowers by the I2 meristem in an apparently helical phyllotaxis (Endress 2010; Tucker 1987). In any case, our observations, based on morphology and on the expression pattern of meristem genes, do not apparently show important differences between the soybean inflorescence and the compound racemes of other legumes such as pea or Medicago (Benlloch et al. 2015; Berbel et al. 2012; Cheng et al. 2018).

### The role of *Dt1* and *GmAP1*s in the soybean genetic inflorescence network

Our results show that *Dt1/GmTFL1b* is expressed in the I1 meristem and its stem vasculature, whereas *GmAP1* is expressed in floral meristems and floral primordia (Fig. 5). In general, this is in agreement with previous descriptions of the expression of these genes in soybean (Chi et al. 2011; Liu et al. 2016; Yue et al. 2021). This expression supports that, as proposed, these two genes regulate meristem identity in soybean, *Dt1/GmTFLb* regulating determination and I1 meristem identity and *GmAP1* regulating floral identity, similarly to their homologs in pea and Medicago *DET/PsTFL1a*, *MtTFL1*, *PIM/PsAP1* and *MtPIM/MtAP1* (Benlloch et al. 2015; Cheng et al. 2018).

The *Dt1* expression pattern that we observed shows some slight differences to the reported *Dt1* expression pattern (Liu et al. 2016). We detected *Dt1/TFL1b* expression only in the center of the I1 meristem, not in the upper meristem layers, similar to the expression reported for its homologs in Medicago, Arabidopsis and *Antirrhinum majus* (Bradley et al. 1997, 1996; Cheng et al. 2018; Liu et al. 2016; Serrano-Mislata et al. 2016). This pattern resembles that observed by Liu et al. (2016) for *Dt1* though they also detected expression extending to outer layers in the SAM of the wild-type plant. Maybe this difference could be due to differences in the developmental stages of the soybean shoot apices analyzed.

The expression of *GmAP1a*, one of the four soybean *AP1* homologs (Chen et al. 2020), does not differ from that of *AP1*-like genes from pea and Medicago (Berbel et al. 2001; Cheng et al. 2018; Chi et al. 2011). It is interesting that the floral phenotype of the loss-of-function mutant of the four soybean *AP1* genes causes a significantly weaker phenotype than that of the *ap1* mutants from pea or Medicago. Thus, while in the pea and Medicago *ap1* mutants, the floral meristems exhibit inflorescence traits, proliferating and producing several flowers with organ defects (Benlloch et al. 2006; Taylor et al. 2002), the quadruple soybean *ap1* mutant only shows floral organ defects (Chen et al. 2020). The origin of this weaker phenotype in soybean is intriguing. A possibility could be redundancy. Nevertheless, the phylogenetic analysis of legume AP1 proteins in Chen et al. (2020) does not suggest the existence of other possible soybean *AP1* genes, apart from the four *AP1* genes presented in that study. Then, if the mild phenotype of the soybean *ap1* quadruple mutant were due to redundancy, this should probably be explained by an additional non-*AP1* gene that would be playing part of the AP1 function. The ClustalW analysis also in that study, including the four soybean AP1 proteins and Arabidopsis AP1, does not point to any obvious change leading to a different function of the soybean AP1 proteins. Finally, another possibility would be that some of the individual *AP1* genes in the soybean quadruple mutant had retained part of their function. Elucidating the origin of the phenotype of the quadruple soybean *ap1* mutant is an interesting question that deserves further study.

### *Dt2* and the specification of I2 meristem identity in soybean

The soybean *Dt2* gene is a homolog of pea *VEG1/PsFULc* and Medicago *MtFULc* (Supplementary Fig. S3), which are expressed in the meristems at the axils of leaves of the inflorescence SAM, which are required for I2 meristem identity (Fig. 6; Berbel et al. 2012; Cheng et al. 2018). Though the expression of *Dt2* at the soybean SAM has been described, to our knowledge not a clear description of *Dt2* expression in the soybean inflorescence is available (Liang et al. 2022; Liu et al. 2016). In the inflorescence apex, we observed strong *Dt2* expression in axillary meristems directly produced by the I1 SAM. These meristems later produce floral meristems, exhibiting strong *GmAP1a* expression, clearly showing that they correspond to I2 meristems (Fig. 6). This shows that *Dt2* is expressed in I2 meristems and supports that, similar to its pea and Medicago homologues, *VEG1/PsFULc* and *MtFULc*, respectively, *Dt2* specifies I2 meristem identity.

The soybean plants bearing the *Dt2*-dominant mutation show a semi-determinate phenotype, where the I1 SAM becomes a terminal inflorescence (Liu et al. 2016; Zhong and Kong 2022). This *Dt2* gain-of-function mutation leads to ectopic *Dt2* expression in the I1 SAM, which represses *Dt1* expression, and causes the conversion of the indeterminate I1 into a terminal raceme (Liu et al. 2016; Ping et al. 2014). This phenotype closely resembles that of the soybean loss-of-function *dt1/gmtfl1b* mutant (Liu et al. 2010; Tian et al. 2010), and of the *det/pstfl1a* and *mttfl1* mutants from pea and Medicago, respectively (Berbel et al. 2001; Foucher et al. 2003). The inflorescence apices of *det/pstfl1a* and *mttfl1* mutants form a terminal I2 inflorescence because of the ectopic expression in the I1 SAM of *VEG1/PsFULc* and *MtFULc*, which specify I2 meristem identity (Berbel et al. 2012; Cheng et al. 2018). In the same way, the presence of *Dt2* in the I1 meristem of the soybean mutant (Liu et al. 2016; Ping et al. 2014) may similarly induce the conversion of the I1 meristem into a terminal I2 inflorescence. This would support a role for *Dt2/GmFULc2* in specifying I2 meristem identity, analogous to the function of *VEG1/PsFULc* and *MtFULc* in pea and *Medicago*, respectively (Fig. 6; Berbel et al. 2012; Cheng et al. 2018).

Loss-of-function mutations in the *VEG1/PsFULc* and *MtFULc* genes, the pea and the Medicago *Dt2* homologues give rise to vegetative plants that never flower because their secondary inflorescences, I2s, are replaced by I1 vegetative primary inflorescences (Berbel et al. 2012; Cheng et al. 2018). While the characterized *Dt2* knockout CRISPR lines show plant architecture defects, such as delayed flowering and maturity, plant height, and increased node and branch numbers, they do not show a vegetative phenotype as that of *veg1/psfulc* and mtfulc pea and Medicago mutants. This does not support a role of *Dt2* on I2 meristem identity (Liang et al. 2022) but it is possible that there is functional redundancy between *Dt2/GmFULc2* and its paralog *GmFULc1* in the specification of I2 meristem identity. It would be important to characterize the inflorescence phenotype of the *dt2/gmfulc2 gmfulc1* double mutant.

### Soybean inflorescence genetic network

In pea and Medicago, a genetic network for specification of identity of the meristems in the inflorescence apex is well established. In this network, both *DET/PsTFL1a* and *MtTFL1*, expressed in the I1 meristem, specify I1 identity; *VEG1/PsFULc* and *MtFULc*, expressed in the I2 meristem, specify I2 meristem identity; and *PIM/PsAP1* and *MtAP1*, expressed in the floral meristem, specify floral identity (Fig. 6). Expression domains of these genes are maintained by a network of reciprocal repression where, for instance, VEG1/MtFULc represses the expression of *DET/MtTFL1* and *PIM/MtAP1* in the I2 meristem, and DET/MtTFL1 and PIM/MtAP1 repress *VEG1/MtFULc* (Fig. 6; Benlloch et al. 2015; Berbel et al. 2012; Cheng et al. 2018). Expression of these genes is complementary in the inflorescence apex of pea and Medicago (Fig. 6). The complementary expression patterns of *Dt1*, *Dt2* and *GmAP1a* in the soybean inflorescence apex closely mirror that of their pea and Medicago homologs (Fig. 6). Moreover, it has also been shown that there is reciprocal repression between these genes in soybean (Liang et al. 2022; Liu et al. 2016; Yue et al. 2021; Zhang et al. 2019). These strong similarities suggest that a gene regulatory network equivalent to that controlling meristem identity in the inflorescence of pea and Medicago is conserved in soybean inflorescence development.

In summary, our study defines the expression pattern of *Dt1/GmTFL1b*, *Dt2/GmFULc2* and *GmAP1a* in the soybean inflorescence, helping to understand how these genes regulate soybean inflorescence development and its growth habit. This knowledge contributes to our understanding of soybean reproductive development and provides a basis for future biotechnological strategies to enhance yield.

## Materials and methods

### Plant material and growth conditions

Soybean plants (*Glycine max*, Williams82—an indeterminate genotype) were grown in 12 cm diameter pots at 22 °C during the day and 18 °C during the night under a long-day (LD) photoperiod (16-h light/8-h darkness). When required, natural light was supplemented with lighting [400W Phillips HDK/400 HPI (R)(N)] to maintain the LD photoperiod. Plants were irrigated periodically using Hoagland N◦1 solution supplemented with oligoelements.

### Phylogenetic analysis

For the phylogenetic tree of the AP1/FUL genes, the deduced amino acid sequences were aligned using the CLUSTALW tool in MACVECTOR 18.5software (MacVector http://www.macvector.com/) and further refined by hand. The evolutionary distances were computed using the p-distance method and a neighbor joining best tree was estimated using systematic tie-breaking and rooted to AtAGL6, an Arabidopsis MADS transcription factor basal to the AP1/FUL clade. The model was accepted based on the high consistency of the resulting topologies with respect to previously published clades and genes.

### Scanning electron microscopy

Vegetative and inflorescence apices were fixed in FAE (50% ethanol, 3.7% formaldehyde, 5% glacial acetic acid) at 4 °C overnight in the dark. Samples were dehydrated with ethanol and critical point dried in liquid CO_2_ (CPD300; Leica, Wetzlar, Germany). Dried samples were sputtercoated with argon–platinum plasma at a distance of 6–7 cm and 45 mA intensity for 15 s in a sputtering chamber (Leica Microsystems EMMED020). Scanning electron microscopy (SEM) micrographs were acquired using an AURIGA compact FIB-SEM (Zeiss, http://www.zeiss.com/) at EHT = 1–2 kV.

### In situ hybridization

RNA in situ hybridization with digoxigenin-labeled probes was performed on 8-μm longitudinal paraffin sections of soybean shoot apices as described previously (Ferrándiz et al. 2000). RNA antisense and sense probes were generated using, as substrate, specific fragments of *Dt1/GmTFL1b*, *Dt2* and *GmAP1a*, amplified by PCR from soybean inflorescence cDNA and cloned into the pGEM-T Easy vector (Promega). Information on the primers used to generate the cDNA fragments for probes is provided in Supplementary Table S1. The sequences of *Dt1/GmTFL1b*  (Glyma.19G194300; Tian et al. 2010), *GmAP1a* (Glyma.16G091300; Chi et al. 2011; Yue et al. 2021), and *Dt2/GmFULc2* (Glyma.18G273600; Ping et al. 2014), whose encoded proteins share 88.75%, 86.21% and 67.69% amino acid identity with the proteins encoded by the pea genes *DET/PsTFL1a*, *PIM/PsAP1*, and *VEG1/PsFULc* (Berbel et al. 2012; Hofer et al. 1997; Taylor et al. 2002), respectively, were retrieved from the soybean genome database (Soybean Knowledge Base, SoyKB). *For Dt1/GmTFL1b*, *Dt2/GmFUL3b* and *GmAP1a*, transcription of antisense and sense probes was carried out with SP6 or T7 polymerases, after linearizing with NcoI or SalI, respectively. Inflorescence apex sections  were hybridized with sense probes for negative controls (Supplementary Fig. S4). Signal was viewed as a purple precipitate under a light microscope.

## Supplementary Information

Below is the link to the electronic supplementary material.

Supplementary file 1 (PDF 4463 KB)

## Data Availability

All biological materials are available from the corresponding author upon request. Supporting data not included in the main text of the article are available in the additional information as supplementary files.

## Declarations

### Generative AI usage

During the preparation of this work, the authors utilized ChatGPT for the purpose of improving the language. After using this tool/service, the authors carefully reviewed and revised the content where necessary, and take full responsibility for the final version of the publication.
