# Supplementary material for: A model for soybean inflorescence architecture based on morphological and gene expression analysis
Source: Planta. 2025 Nov 13;263(1):5. doi: 10.1007/s00425-025-04864-1 (PMC12615538; doi:10.1007/s00425-025-04864-1)
Supplement: Supplementary file 1 — (PDF 4463 KB) [file 425_2025_4864_MOESM1_ESM.pdf]

Supplementary data Pozo-Muñoz et al.

Title: Morphological and gene expression analysis of the soybean inflorescence suggests a genetic network governing its architecture

Journal: Planta

Authors: Francisca Pozo-Muñoz[1], Ana Berbel[1], Fanjiang Kong[2]* and Francisco Madueño[1]*

Affiliation:
[1] Instituto de Biología Molecular y Celular de Plantas, CSIC-UPV, Campus de Vera, 46022 Valencia, Spain.

[2] Guangdong Provincial Key Laboratory of Plant Adaptation and Molecular Design, Innovative Center of Molecular Genetics and Evolution, School of Life Sciences, Guangzhou University, Guangzhou 510006, China.

Figure S1: Compound inflorescence architecture in pea.

Figure S2: Inflorescence architecture in soybean.

Figure S3. Phylogenetic tree from predicted amino acid sequences from AP1/FUL family.

Figure S4: Negative controls of *in situ* hybridization experiments with *Dt1*, *Dt2*, *GmAP1a*.

Table S1. List of primers used for *in situ* hybridization analyses.

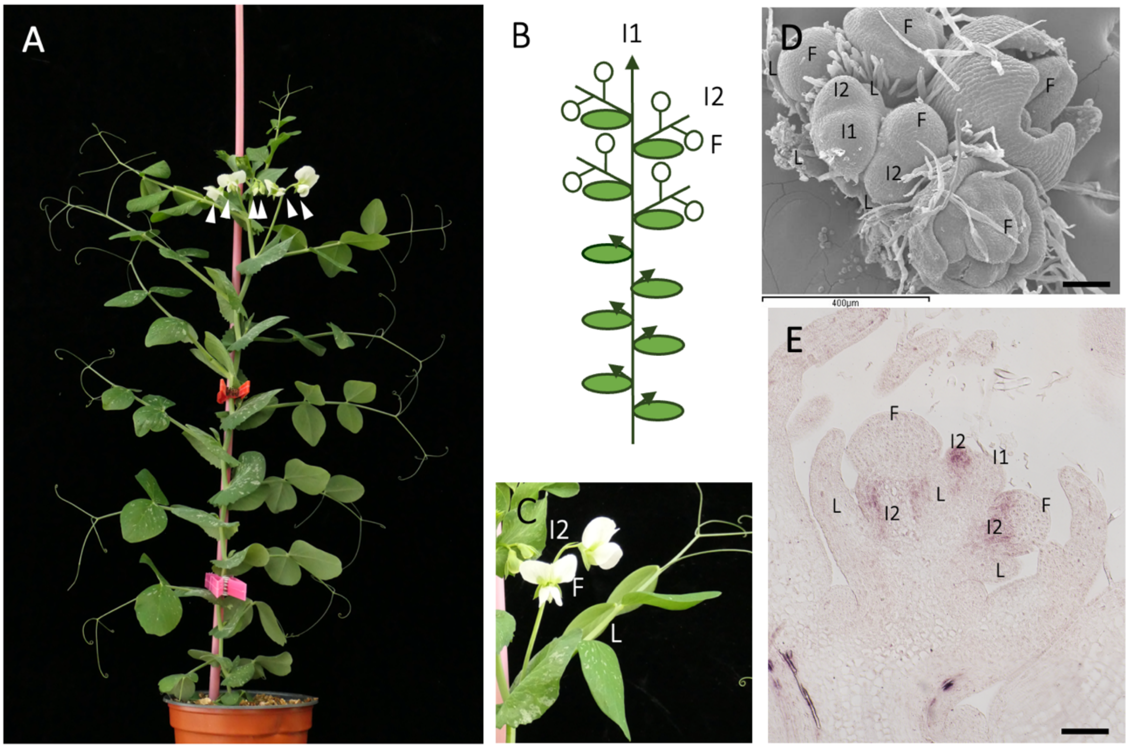

Figure S1. Compound inflorescence architecture in pea. A Pea adult plant, with three secondary inflorescences (I2s), each of them with two flowers (arrowheads). **B** Diagram of a pea adult plant (arrows, meristems; green ovals, leaves; closed circles, flowers). **C** Close-up of a pea I2, subtended by a leaf, bearing two flowers. **D** SEM image of a pea inflorescence apex. **E** Section of a pea inflorescence apex hybridized with the I2 specific gene *VEG1/PsFULc*. I1, primary inflorescence; I2, secondary inflorescence; F, flower; L, leaf. Scale bars: 100mm.

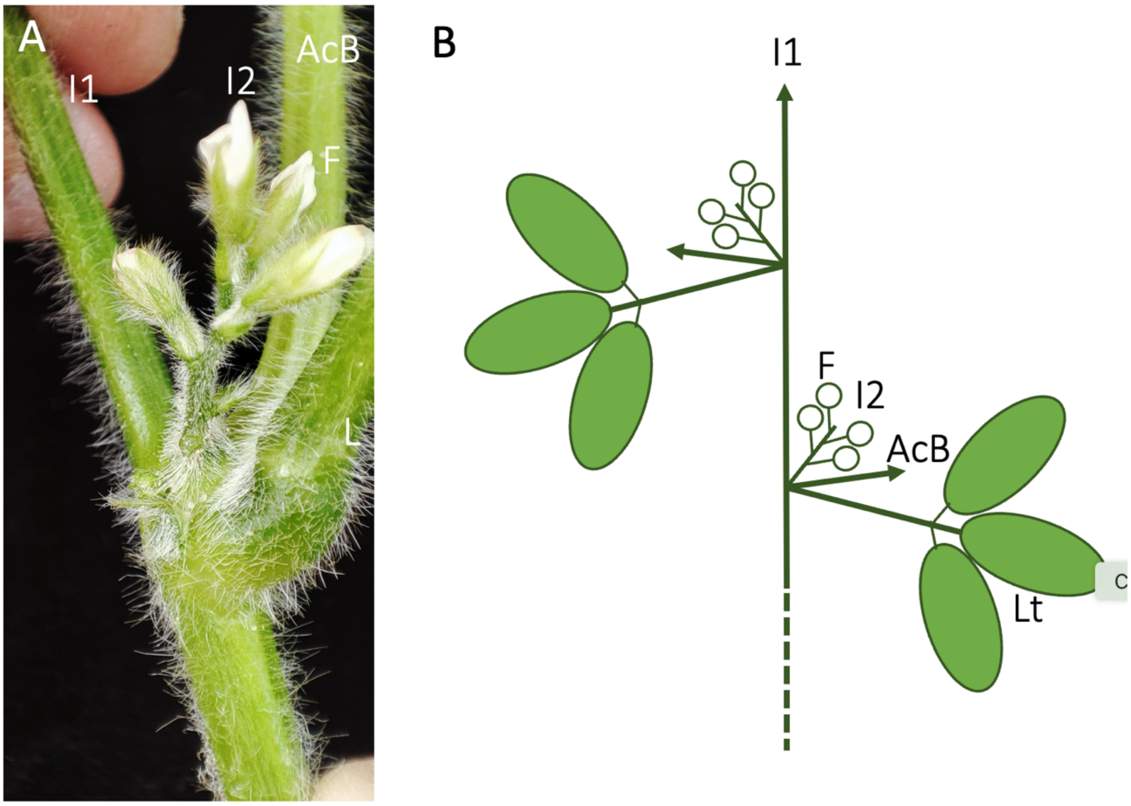

Figure S2. Inflorescence architecture in soybean. A Image of a soybean reproductive node bearing an axillary inflorescence with four flowers and an accessory branch. **B** Diagram of a segment of the primary reproductive stem of soybean with two reproductive nodes. Lt, trifoliate leaf; I1, primary inflorescence; I2, secondary inflorescence; F, flower; AcB: accessory branch.

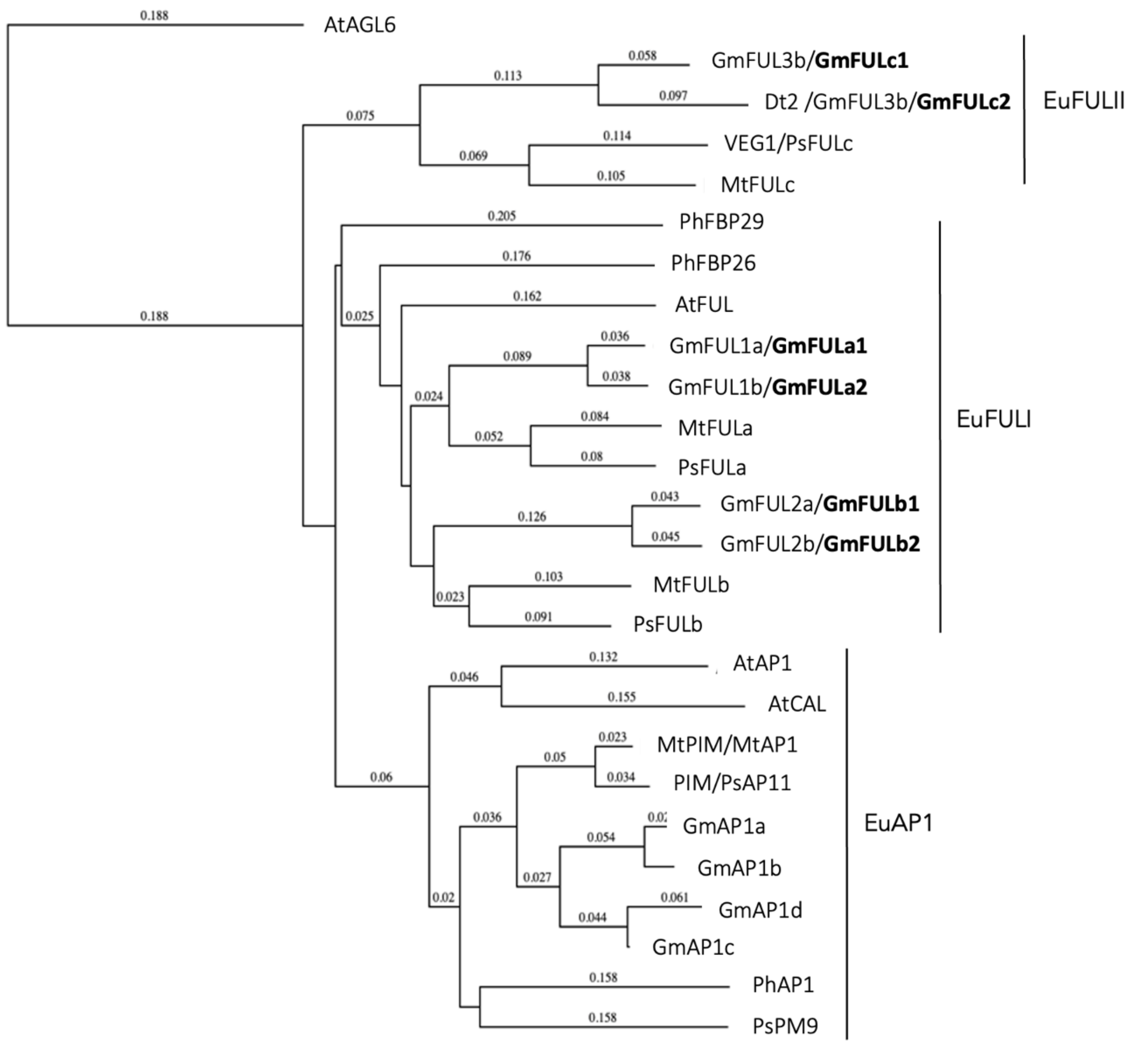

Figure S3. Phylogenetic tree from predicted amino acid sequences from AP1/FUL family. DET2 is phylogenetically close to VEG1/PsFULc and MtFULc. AtAGL6 (AAC06173), GmFUL3a/GmFULc1 (Glyma08g27680), Dt2 /GmFUL3b/GmFULc2 (Glyma16G091300), VEG1/PsFULc (AFI08225.1), MtFULc (Medtr7g016630.1), PhFBP29 (AAK21258.1), PhFBP26 (AAF19164.1), AtFUL (sp|Q38876.1|AGL8_ARATH), GmFUL1a/GmFULa1 (Glyma04g31847), GmFUL1b/GmFULa2 (Glyma06g22650), MtFULa (Medtr2g461760.1), PsFULa (AAX69065.1), GmFUL2a/GmFULb1 (Glyma05g07380), GmFUL2b/GmFULb2 (Glyma17g08890), MtFULb (Medtr4g109830.1), PsFULb (AFI08227.1), AtAP1 (AAG50679.1), AtCAL (AAG50679.1), MtPIM/MtAP1 (Medtr8g066260.1), PIM/PsAP1 (AAL66379.1), GmAP1a (Glyma.16G091300), GmAP1b (Glyma.08G269800), GmAP1c (Glyma.01G064200), GmAP1d (Glyma.02G121600), PhAP1 (QEU52638.1), PsPM9 (Psat02G0257600-T1). At, Arabidopsis thaliana; Gm, Glycine maxima; Mt Medicago truncatula; Ph, Petunia hybrida. New names suggested for GmFUL proteins are highlighted with bold type.

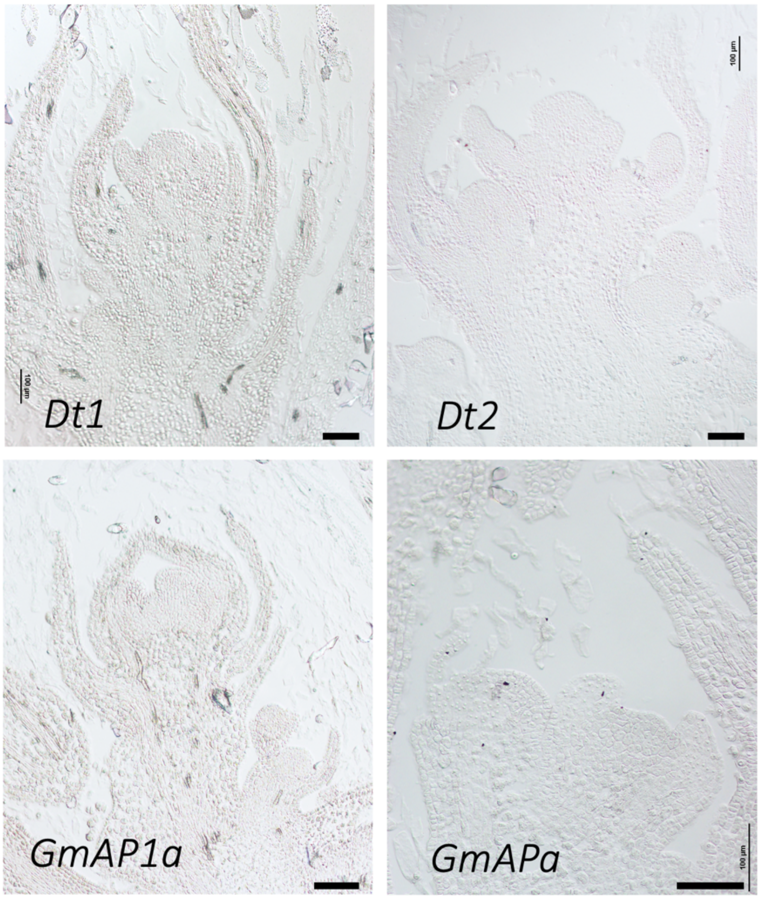

Figure S4. Negative controls of *in situ* hybridization experiments with *Dt1*, *Dt2*, *GmAP1a*. For control, sections of soybean inflorescence apices were hybridized with sense probes of each of these genes. Upper part, *Dt1* and *Dt2* sense probes tested on inflorescence apices. Lower part left, *GmAP1a* sense probe tested on an inflorescence apex. Lower part right, *GmAP1a* tested on an I2 with two floral buds. Scale bars: 100mm.

Table S1. List of primers used for *in situ* hybridization analyses.

Primer ID	Primer sequence
GmDt2_Fw	TGGAGCTAACAATGTCGAATC
GmDt2_Rev	ACCCGTTTCTTCCACTGGTG
GmAP1a_Fw	TATGAAAGGTATGCCTATGCAGAG
GmAP1a_Rev	ACTCAACGCTTAATTAGTTCAGATAC
GmDt1_Fw	TGTTCAAGCAAAAGCGTAGAC
GmDt1_Rev	AAGATCACCTCATAGTAGTACC
